# Electroencephalography Enables Continuous Decoding of Hand Motion Angles in Polar Coordinates

**DOI:** 10.34133/cbsystems.0469

**Published:** 2026-01-12

**Authors:** Xiaohan Lu, Yifeng Chen, Zhiying Li, Jinqiu Zhao, Yijie Zhou, Dongrui Wu, Mingming Zhang

**Affiliations:** ^1^ Shenzhen Key Laboratory of Smart Healthcare Engineering, Department of Biomedical Engineering, Southern University of Science and Technology, Shenzhen 518055, China.; ^2^ Medical School of Tianjin University, Tianjin 300072, China.; ^3^ Haihe Laboratory of Brain-Computer Interaction and Human-Machine Integration, Tianjin 300392, China.; ^4^ Shenzhen Huazhong University of Science and Technology Research Institute, Shenzhen 518000, China.

## Abstract

Hand movements in task space are typically represented using either Cartesian or polar coordinate systems. While Cartesian coordinates are commonly used in electroencephalography (EEG)-based brain–computer interface (BCI) studies, polar coordinates offer a more natural representation for circular motion by directly encoding angular information. This study investigates the feasibility of continuous decoding of hand motion angles in polar coordinates using EEG signals. In the paradigm, human participants engaged in bimanual circular tracing with a fixed radius while their EEG signals were recorded. To evaluate the feasibility of this approach, 6 deep learning models, including commonly used EEGNet, DeepConvNet, and ShallowConvNet, and their variants incorporating long short-term memory (LSTM) layers, were employed. Performance was assessed using mean squared error (MSE), mean absolute error (MAE), and correlation coefficient (CC) between decoded and actual angles. Across 8 participants, all 6 models significantly outperformed the chance level (*P* < 0.01), with the best model achieving an MSE of 1.012 rad^2^, an MAE of 0.627 rad, and a CC of 0.895. These results demonstrate the feasibility of continuous angular decoding of circular hand motion in polar coordinates using EEG signals. This approach offers a promising alternative to traditional Cartesian-based decoding methods, particularly for applications involving circular or rotational movements.

## Introduction

Brain–computer interface (BCI) records and decodes brain signals, enabling interaction between the brain and external devices, making it particularly beneficial for patients with neurological disorders and motor function loss [1–5]. BCIs can be categorized into invasive [6] and noninvasive types [7]. Noninvasive BCIs provide several benefits compared to invasive systems, including lower costs [8], no need for surgery [9], and reduced risk of complications [10]. Given these practical advantages, electroencephalography (EEG), as a type of noninvasive BCI, has become the focus of numerous studies.

In most EEG-based BCIs, sensorimotor rhythm (SMR) paradigm is commonly employed [11–17]. Within this paradigm, the execution or imagination of movements involving the hands, feet, tongue, and other body parts induces modulations in EEG signals, known as event-related desynchronization/synchronization (ERD/ERS) [11]. By classifying EEG patterns, BCI systems can translate brain activity into control signals, allowing users to operate external devices directly with their brain. For instance, Edelman et al. [12] designed a 4-classification paradigm and achieved high-quality neural control of a robotic device for random target tracking. However, the SMR paradigm presents 2 key shortcomings. First, it offers only limited discrete categorical information, which constrains its ability to represent the continuous nature of movement. Second, the mapping of body parts to control commands lacks both naturalness and alignment with the user’s intuitive sense of control [17].

To overcome the limitations of SMR, several studies [18–22] have successfully decoded continuous limb kinematic information from EEG. For example, Robinson et al. [18] reconstructed single-hand position and velocity along the horizontal axis. Chen et al. [19] decoded bimanual movement trajectories in one-dimensional (1D) space. Úbeda et al. [20] achieved continuous decoding of 2D hand motion. Korik et al. [21] demonstrated that executed and imagined arm movements in 3D space can also be decoded continuously. Similarly, Sosnik and Ben-Zur [22] successfully decoded both executed and imagined trajectories of hand, elbow, and shoulder in 3D space. These studies collectively demonstrate the feasibility of continuously decoding kinematic information from EEG, offering promising applications in motor rehabilitation by enabling more precise monitoring and assessment of patient movements.

Center-out paradigms have been widely used in continuous motor decoding studies to examine the relationship between brain activity and voluntary movement [23–26]. In these paradigms, participants are asked to perform a movement that involves starting from a central point (often referred to as the “center”) and reaching or moving toward one or more targets placed around that central point. For instance, Zeng et al. [23] instructed participants to perform the classic center-out task with 4 targets on either the *x* axis or the *y* axis to decode their movement trajectories. Wang et al. [24] improved this paradigm by positioning 4 orthogonal targets at 45° angles to the Cartesian coordinate axes to enhance decoding performance. Úbeda et al. [25] expanded the center-out paradigm from 4 to 8 targets. Hosseini and Shalchyan [26] introduced randomly distributed targets, each maintaining a minimum angular separation of 20° from the horizontal and vertical axes, to test the decoder’s generalizability. Bradberry et al. [27] further extended the original paradigm from 2D to 3D space. These studies highlighted the versatility and effectiveness of the center-out paradigm in EEG-based motor decoding. However, since the movements performed by participants in this paradigm primarily involved straight-line motions, a substantial gap remains between these motions and actual limb movements, which limits its practical importance.

To bridge this gap, some studies [28–31] have utilized more complex paradigms than the center-out paradigm and achieved promising results. Lv and Li [28] instructed the subjects to move their hands in 4 directions while performing a 2D polyline drawing task and decoded the movement velocities. Mondini et al. [29] employed a more complex paradigm in which participants tracked a moving snake on a screen with a robotic arm. Kim et al. [30] created a 3D movement trajectory by folding an infinity symbol at a specific angle and reconstructed executed and imagined hand motions. Wang et al. [31] guided subjects to execute sign language sentences and continuously decoded the 3D coordinates of upper limb joint points using their specialized model. The motion paradigms employed in these studies more closely mimic real-world scenarios by incorporating complex and diverse movement trajectories, in contrast to the structured and simplified center-out paradigm.

Although diverse paradigms have emerged, the Cartesian coordinate system remains the preferred choice for the vast majority of continuous movement decoding research [32–38]. While Cartesian coordinates effectively model straight-line motions, such as those in the center-out paradigm, they are less efficient for representing circular or rotational movements, which are common in real-life scenarios. In contrast, polar coordinates provide a more natural and compact representation for circular movements. Defined by a radius and an angle from an origin, they directly encode angular information. This inherent alignment with rotational kinematics could simplify the decoder’s task of learning such patterns, potentially improving accuracy and reducing computational complexity. However, to our knowledge, no prior studies have explored the continuous decoding of whole-hand motion in polar coordinates from EEG.

In light of this issue, we designed a circular motion paradigm to investigate the feasibility of decoding polar coordinates from EEG signals, focusing specifically on polar angles defined in the task space. The circular geometry intrinsically aligns with polar coordinate systems, providing an ideal framework for angular decoding. Moreover, compared to conventional center-out paradigms, this design provides greater kinematic complexity through continuous curved paths that better approximate naturalistic movement patterns. By employing this paradigm, we aim to investigate the unexplored potential of polar coordinates in continuous motor decoding. The primary contributions of this study are as follows: (a) To the best of our knowledge, this is the first instance where angles of circular hand motion in polar coordinates have been continuously decoded from EEG signals; (b) the feasibility of this approach was experimentally demonstrated on human subjects using multiple deep learning models.

## Materials and Methods

### Participants and experimental paradigm

Eight healthy human subjects (6 males and 2 females), aged 20 to 26 years (mean 21.08 ± 1.62), participated in this experiment. All volunteers were healthy without brain diseases and naive to BCI. They signed informed consent and were confirmed to be right-handed by the Edinburgh Handedness Inventory. This study was approved by the Ethics Committee of the Southern University of Science and Technology (no. 20230049).

**Fig. 1.**
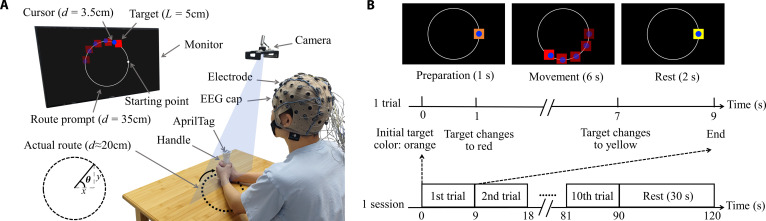
Experimental paradigm. (A) Experimental setup including an EEG cap with 32 electrodes, a monitor, a handle with an AprilTag, and a camera for location recording. The content displayed on the monitor includes a circular cursor, a square target, and a circular route prompt. Note that the translucent cursors and targets in the figure are only used to represent the motion process; they do not appear on the monitor in the actual experiment. (B) Trial and session sequence.

The experimental setup is shown in Fig. 1A. Subjects were seated in front of a desk. A 43-inch display monitor was placed at a comfortable viewing distance of approximately 1.5 m. Subjects were instructed to perform a continuous visuomotor tracking task shown on the screen. Several visual cues were provided, including a square target, a circular cursor representing hand position, and a circular route as a reference. The subjects were required to align the cursor with the target as accurately as possible. The target started at the rightmost point of the circular route, moved clockwise at a constant speed, and returned to its starting position after one full rotation. Subjects controlled the cursor by moving a handle with both hands. An AprilTag [39] was attached to the top of the handle for position tracking. A camera mounted approximately 60 cm above the desk captured the position of the AprilTag. The actual movement trajectory of their hands had an approximate diameter of 20 cm. The continuous polar angle (θ) of hand motion in polar coordinates was the decoding objective of this study.

As shown in Fig. 1B, the experiment consisted of 20 identical sessions separated by 30-s rest periods. Each session included 10 trials. Each trial consisted of 1 s of preparation, during which the target was orange; 6 s of movement, during which the target changed to red; and 2 s of rest, during which the target was yellow. Subjects were asked to minimize eye blinks and body movements during preparation and movement periods. A video illustrating the experimental paradigm is provided as supplementary material (Movie S1).

### Data acquisition and preprocessing

EEG signals of 32 channels were continuously recorded at a sampling rate of 256 Hz by the g.HIAMP 256 bio-signal amplifier (g.tec medical engineering GmbH, Austria). The electrodes were arranged according to the international 10–10 system. The ground was placed at the AFz. The impedance of all electrodes was kept below 30 kΩ. A 50-Hz notch filter was applied to reduce power line interference. Additionally, the camera recorded the positional data (in Cartesian coordinates), which were then used to calculate the polar angles.

The EEG data were preprocessed by the open-source toolbox EEGLAB [40]. First, the EEG data were bandpass filtered from 0.1 to 30 Hz using a zero-phase finite impulse response filter. Next, bad channels were selected manually by inspecting time-domain signals, frequency spectra, and topographic maps. Once identified, bad channels were interpolated using spherical spline interpolation to estimate their signals based on neighboring electrodes. Then, the data were re-referenced by the common average reference (CAR). Subsequently, the independent component analysis (ICA) was performed on each session separately to identify and remove ocular artifacts. Since ICA performs better on 1-Hz high-pass filtered data, the data were filtered at this cutoff frequency before ICA decomposition. The resulting ICA weights were then applied to the data without 1-Hz high-pass filtering. This approach ensured the quality of ICA while preserving low-frequency information. Ocular artifact components were automatically labeled by EEGLAB’s built-in function “ICLabel”, with a threshold of 0.85. The number of removed components varied across subjects and sessions, but was empirically limited to a maximum of 3 to balance artifact removal with neural signal preservation. Finally, the continuous EEG data were segmented into trials, and baseline correction was performed by subtracting the average voltage of data from −1 to 0 s relative to movement onset for each epoch of movement to eliminate the drift.

For positional data, the sampling rate fluctuated between 400 and 600 Hz due to the unstable frame rate of the camera. Therefore, interpolation was performed to uniformly increase the sampling rate to 1,000 Hz. Besides, in rare cases, the camera would momentarily lose track of the target, causing the recorded position to deviate substantially from the true position. To address this, all trajectories were visually inspected and outliers were replaced with the average of the preceding and following points. Since the positional data were recorded in Cartesian coordinates (*x, y*), the polar angle *θ* was calculated using the following formula (*θ* = 0 if *x* ≥ 0 and *y* = 0; *θ* = 0.5π if *x* = 0 and *y* > 0; *θ* = 1.5π if *x* = 0 and *y* < 0):

$$\begin{array}{c} \theta =\left\{\begin{array}{cc} \text{arctan}\left(\frac{y}{x}\right) & \text{if}\;x\;>\;0\;\text{and}\;y\;>\;0\\ \pi +\text{arctan}\left(\frac{y}{x}\right) & \text{if}\;x\;<\;0\;\text{and}\;y\;\neq 0\\ 2\pi +\text{arctan}\left(\frac{y}{x}\right) & \text{if}\;x\;>0\;\text{and}\;y\;<0 \end{array}\right. \end{array}$$ (1)

The resulting polar angle was constrained to the range [0, 2π) instead of [−π, π) to prevent discontinuities that could degrade training results. The angle data were then synchronized to EEG and split into trials based on recorded triggers.

Finally, both the EEG and angle data were down-sampled to 100 Hz, and EEG was then standardized individually for each channel using the following formula:

$$s_{n}\left[t\right]=\frac{v_{n}\left[t\right]-a_{n}\left[t\right]}{\sigma_{n}\left[t\right]}$$ (2)

where *v_n_*[*t*] is the EEG amplitude at channel *n* (ranging from 1 to 32) and time *t*, *a_n_*[*t*] is the average of EEG amplitude at channel *n*, and *σ_n_* is the standard deviation of EEG amplitude at channel *n*.

### Network architecture

In this study, 6 deep learning networks, including 3 common convolutional neural networks (CNNs) and their corresponding long short-term memory (LSTM)-hybrid architectures, were applied. The 3 CNNs we selected have different levels of structural complexity to ensure a comprehensive evaluation. First, as the simplest architecture in this study, ShallowConvNet [41] sequentially contains a temporal convolutional layer and a spatial filtering layer. DeepConvNet [41] incorporates a deeper architecture with 5 convolutional layers. EEGNet [42] employed more diversified convolutional layers, comprising 2 blocks: The first block includes 2D and depthwise convolutions; the second block is based on separable convolution. All networks shared common components such as dropout and batch normalization. The softmax activation function in the output layer was replaced with a linear one to perform regression tasks.

**Fig. 2.**
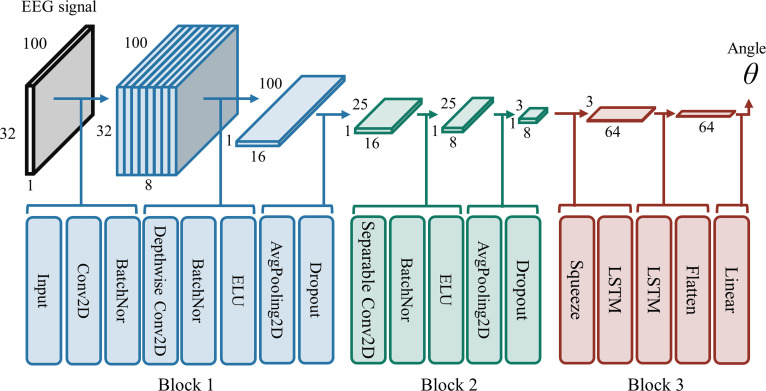
Illustration of the EEGNet + LSTM hybrid architecture. The first 2 blocks correspond to EEGNet. The final block contains 2 LSTM layers, each with 64 units.

However, these CNNs are limited in modeling temporal dependencies in both EEG signals and polar angle data, which are crucial for continuous motion decoding. In contrast, LSTM excels at capturing such dependencies through their gated memory cells and adaptive forgetting mechanisms. The integration of CNN with LSTM layers enables the model to effectively capture both spatial and temporal dynamics, thus enhancing continuous motion decoding [19]. The structure of EEGNet + LSTM hybrid network is shown in Fig. 2. The outputs from the final convolutional layer were reshaped to meet the input requirements of the LSTM module. These reshaped outputs were then passed through the 2 LSTM layers in sequence, a configuration that balanced model capacity against overfitting risk. Similarly, we combined DeepConvNet and ShallowConvNet with LSTM to improve motion decoding.

### Model training and evaluation

#### Dataset construction

The EEG signals used for analysis included the last 0.5 s of the preparation period, the entire movement period, and the first 0.5 s of the rest period in each trial. This approach was taken to ensure that no angle data from the beginning or end of the movement period is lost after applying the sliding window. We ultimately obtained 8 subject-specific datasets, each containing 200 trials. In each subject-specific dataset, all trials were randomly shuffled, followed by systematic sampling to select 20 trials as the test set, with the remaining 180 trials divided into 10 nonoverlapping folds for cross-validation.

Within each fold, the EEG trials were further segmented by sliding window. We set the window size to 32 × 100 (channel × time). The length and moving steps were 1 and 0.05 s, respectively. After discarding incomplete segments at the endpoints, each trial ultimately generated 121 valid windows. The angle data were also separated to synchronize with EEG windows. The 50th sample point (center point) of the angle data in each window was considered as the predicting label for the corresponding EEG segment. The near-uniform circular motion ensured that the center point accurately represented the window’s angular state, with a negligible difference ((0.026 ± 0.023) rad, averaged across all windows) from the mean angle within the same window.

#### Hyperparameters and model training

Hyperparameters such as the number of filters in the first convolutional layer, dropout rate, loss function, optimizer, learning rate, batch size, and epoch were consistent across all subjects in the models mentioned above. The LSTM-combined models retained the same configurations as their original CNN counterparts. The final hyperparameters were determined through a manual search, guided by the 10-fold cross-validated performance (test set not involved). This process led to the following configurations: The first convolutional layer had 8, 25, and 40 filters in EEGNet, DeepConvNet, and ShallowConvNet, respectively. Each LSTM layer contained 64 units. Dropout rate was set to 0.2, loss function to mean squared error (MSE), optimizer to Adam, and learning rate to 0.002 without decay. The batch size was set to 1,089 windows (from 9 trials), with a maximum training epoch of 1,000. Early stopping was employed to terminate training if validation loss showed no improvement over 50 epochs. All the models were trained on NVIDIA GeForce RTX 4070 Super GPU and AMD R5 7500F CPU, with CUDA 11.2 using the Tensorflow API.

#### Performance evaluation

Subject-specific models were built and 10-fold cross-validation was applied for performance evaluation. Each fold was evaluated on the test set. The decoding performance was analyzed in terms of the MSE, mean absolute error (MAE), and Pearson correlation coefficient (CC) between decoded and real angles. To establish a chance-level baseline, the same 10-fold cross-validation procedure was used, but with the labels in the training and validation sets randomly permuted prior to model training for each fold. Each model’s decoding performance was then compared against their baseline to validate its feasibility.

## Results and Discussion

### Angle decoding performance

**Fig. 3.**
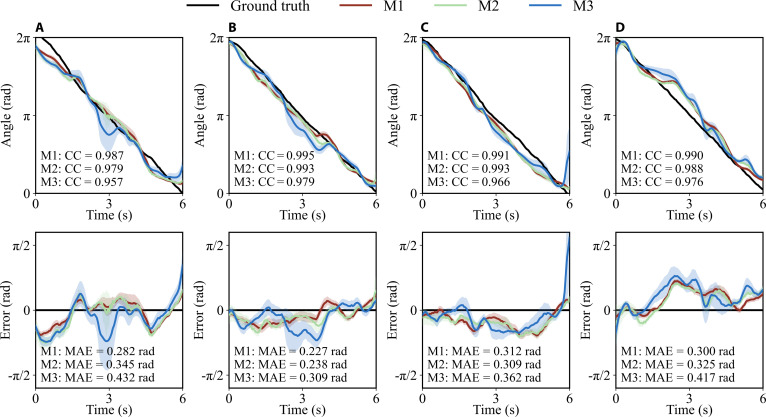
Ten-fold average decoding angles and errors for the first 4 trials in the test set of subject 1. (A) Trial 1. (B) Trial 2. (C) Trial 3. (D) Trial 4. M1 = EEGNet + LSTM. M2 = DeepConvNet + LSTM. M3 = ShallowConvNet + LSTM. The 95% confidence intervals (CIs) are represented by shaded areas, colored to match their corresponding models.

In order to evaluate continuous angle decoding performance, Fig. 3 compares the 10-fold average decoding angles and the ground truth for the first 4 trials in the test set of subject 1. The error for each trial is displayed below the results. The black solid line represents the ground truth, while the red, green, and blue lines correspond to EEGNet + LSTM, DeepConvNet + LSTM, and ShallowConvNet + LSTM, respectively. The average CC and MAE for these 3 models on the given trials are indicated in the figure.

**Fig. 4.**
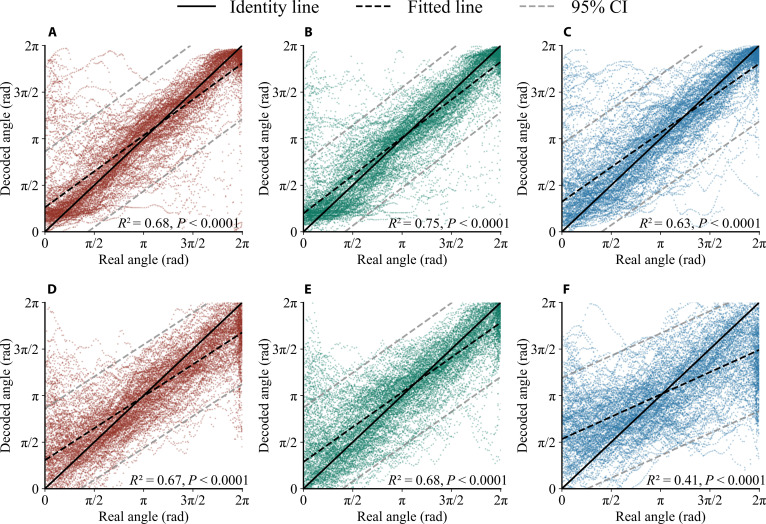
Scatterplot of the actual versus predicted angle. (A) Results of EEGNet + LSTM, (B) DeepConvNet + LSTM, and (C) ShallowConvNet + LSTM. (D) Results of EEGNet, (E) DeepConvNet, and (F) ShallowConvNet.

Fig. 4 presents a scatterplot of the actual versus predicted angle, for a more intuitive assessment of the correlation between actual and predicted angles. The plot is based on the best folds for each subject. The same color indicates that the 2 models share the same CNN architecture. The black solid line serves as an identity line (*y* = *x*). The black dashed line represents the fitted line. The gray dashed lines mark the 95% prediction interval (PI). The coefficient of determination (i.e., *R*^2^) is marked in the figure, with higher values indicating better performance. Among the 6 models, DeepConvNet + LSTM achieved the best results, with *R*^2^ reaching 0.75. All LSTM-combined models achieved higher *R*^2^ compared to their original CNN counterparts.

To quantify the performance analysis, Tables 1 to 3 respectively list the average MSE, MAE, and CC of 6 models across 8 subjects. At the grand-averaged level, EEGNet + LSTM and DeepConvNet + LSTM achieved the best performance, with no significant difference between them (*P* > 0.05). Specifically, EEGNet + LSTM yielded an MSE of (1.054 ± 0.403) rad^2^, MAE of (0.619 ± 0.140) rad, and CC of (0.885 ± 0.049). For DeepConvNet + LSTM, the corresponding values were (1.012 ± 0.436) rad^2^, (0.627 ± 0.152) rad, and (0.895 ± 0.053). In contrast, ShallowConvNet + LSTM underperformed both, with an MSE of (1.327 ± 0.650) rad^2^, MAE of (0.754 ± 0.240) rad, and CC of 0.845 ± 0.098. Notably, all hybrid models outperformed their original CNN counterparts, although the degree of improvement varied. Furthermore, all models except ShallowConvNet demonstrated grand-averaged CC values approaching 0.90. At the subject-specific level, the minimum MSE reached (0.410 ± 0.193) rad^2^, the lowest MAE reached (0.384 ± 0.046) rad, and the CC peaked at (0.971 ± 0.008).

**Table 1.** Mean squared error between the true angles and decoded angles by different models across subjects (mean ± SD). Results for each subject were obtained using 10-fold cross-validation, with units in radians squared.

	Subj.1	Subj.2	Subj.3	Subj.4	Subj.5	Subj.6	Subj.7	Subj.8	Avg.
M1	0.516 ± 0.157	1.010 ± 0.168	0.581 ± 0.093	1.852 ± 0.215	1.160 ± 0.128	1.034 ± 0.169	0.888 ± 0.259	1.387 ± 0.206	1.054 ± 0.403
M2	0.410 ± 0.193	1.001 ± 0.266	0.524 ± 0.111	1.847 ± 0.530	0.983 ± 0.191	1.397 ± 0.242	0.783 ± 0.219	1.152 ± 0.119	1.012 ± 0.436
M3	0.574 ± 0.170	1.637 ± 0.424	0.571 ± 0.105	2.775 ± 0.511	1.180 ± 0.193	1.419 ± 0.270	1.213 ± 0.238	1.246 ± 0.224	1.327 ± 0.650
M4	0.745 ± 0.142	1.201 ± 0.285	0.800 ± 0.117	1.907 ± 0.269	1.253 ± 0.147	1.186 ± 0.064	1.072 ± 0.177	1.319 ± 0.228	1.185 ± 0.335
M5	0.640 ± 0.109	1.148 ± 0.224	0.576 ± 0.093	1.563 ± 0.198	1.061 ± 0.189	1.231 ± 0.134	1.029 ± 0.144	1.258 ± 0.154	1.063 ± 0.304
M6	1.908 ± 0.906	2.839 ± 0.327	1.481 ± 0.170	3.056 ± 0.231	1.957 ± 0.175	2.072 ± 0.554	2.052 ± 0.119	2.480 ± 0.238	2.231 ± 0.489

SD, standard deviation; M1, EEGNet + LSTM; M2, DeepConvNet + LSTM; M3, ShallowConvNet + LSTM; M4, EEGNet; M5, DeepConvNet; M6, ShallowConvNet

**Table 2.** Mean absolute error between the true angles and decoded angles by different models across subjects (mean ± SD). Results for each subject were obtained using 10-fold cross-validation, with units in radians.

	Subj.1	Subj.2	Subj.3	Subj.4	Subj.5	Subj.6	Subj.7	Subj.8	Avg.
M1	0.384 ± 0.046	0.676 ± 0.058	0.484 ± 0.030	0.897 ± 0.071	0.628 ± 0.037	0.636 ± 0.069	0.604 ± 0.071	0.647 ± 0.080	0.619 ± 0.140
M2	0.394 ± 0.096	0.691 ± 0.093	0.490 ± 0.033	0.939 ± 0.148	0.600 ± 0.043	0.715 ± 0.021	0.594 ± 0.062	0.593 ± 0.038	0.627 ± 0.152
M3	0.471 ± 0.084	0.910 ± 0.167	0.521 ± 0.046	1.275 ± 0.176	0.682 ± 0.063	0.803 ± 0.104	0.764 ± 0.072	0.606 ± 0.061	0.754 ± 0.240
M4	0.567 ± 0.059	0.821 ± 0.102	0.631 ± 0.046	1.071 ± 0.077	0.809 ± 0.058	0.812 ± 0.027	0.784 ± 0.061	0.752 ± 0.062	0.781 ± 0.140
M5	0.521 ± 0.040	0.789 ± 0.093	0.536 ± 0.039	0.906 ± 0.067	0.722 ± 0.083	0.783 ± 0.041	0.737 ± 0.056	0.723 ± 0.052	0.715 ± 0.121
M6	1.072 ± 0.329	1.392 ± 0.106	0.930 ± 0.049	1.414 ± 0.069	1.046 ± 0.068	1.130 ± 0.161	1.096 ± 0.034	1.174 ± 0.068	1.157 ± 0.157

SD, standard deviation; M1, EEGNet + LSTM; M2, DeepConvNet + LSTM; M3, ShallowConvNet + LSTM; M4, EEGNet; M5, DeepConvNet; M6, ShallowConvNet

**Table 3.** Pearson correlation coefficient between the true angles and decoded angles by different models across subjects (mean ± SD). Results for each subject were obtained using 10-fold cross-validation.

	Subj.1	Subj.2	Subj.3	Subj.4	Subj.5	Subj.6	Subj.7	Subj.8	Avg.
M1	0.931 ± 0.025	0.895 ± 0.021	0.958 ± 0.015	0.792 ± 0.032	0.882 ± 0.015	0.886 ± 0.021	0.898 ± 0.037	0.834 ± 0.019	0.885 ± 0.049
M2	0.954 ± 0.019	0.895 ± 0.037	0.971 ± 0.008	0.796 ± 0.073	0.910 ± 0.023	0.853 ± 0.019	0.914 ± 0.030	0.863 ± 0.017	0.895 ± 0.053
M3	0.931 ± 0.022	0.778 ± 0.082	0.964 ± 0.011	0.626 ± 0.122	0.890 ± 0.023	0.848 ± 0.030	0.876 ± 0.031	0.845 ± 0.032	0.845 ± 0.098
M4	0.936 ± 0.014	0.881 ± 0.030	0.931 ± 0.014	0.809 ± 0.027	0.884 ± 0.025	0.883 ± 0.014	0.889 ± 0.026	0.867 ± 0.026	0.885 ± 0.037
M5	0.944 ± 0.013	0.886 ± 0.032	0.958 ± 0.009	0.840 ± 0.032	0.914 ± 0.021	0.861 ± 0.029	0.905 ± 0.019	0.879 ± 0.025	0.898 ± 0.038
M6	0.694 ± 0.204	0.485 ± 0.126	0.831 ± 0.035	0.496 ± 0.075	0.752 ± 0.033	0.737 ± 0.096	0.721 ± 0.031	0.729 ± 0.036	0.680 ± 0.116

SD, standard deviation; M1, EEGNet + LSTM; M2, DeepConvNet + LSTM; M3, ShallowConvNet + LSTM; M4, EEGNet; M5, DeepConvNet; M6, ShallowConvNet

**Fig. 5.**
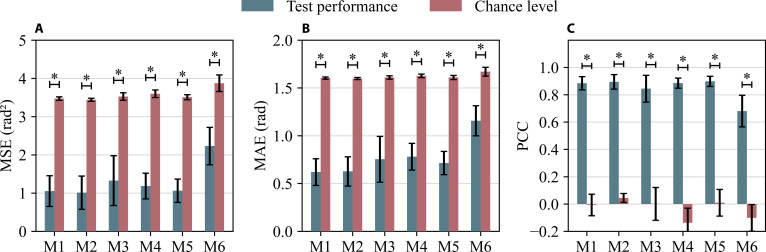
Comparison of test performance and chance level across 6 models. (A) Comparison on mean squared error, (B) mean absolute error, and (C) Pearson correlation coefficient. *P < 0.001.

To further compare the test performance and the chance level in angle decoding, we performed an analysis of variance (ANOVA) to statistically analyze the MSE, MAE, and CC as shown in Fig. 5. The test performance of all models across all metrics was significantly better (*P* < 0.001) than their respective chance levels. This confirms the feasibility of continuous angle decoding from EEG signals across various models.

### Neural representation

**Fig. 6.**
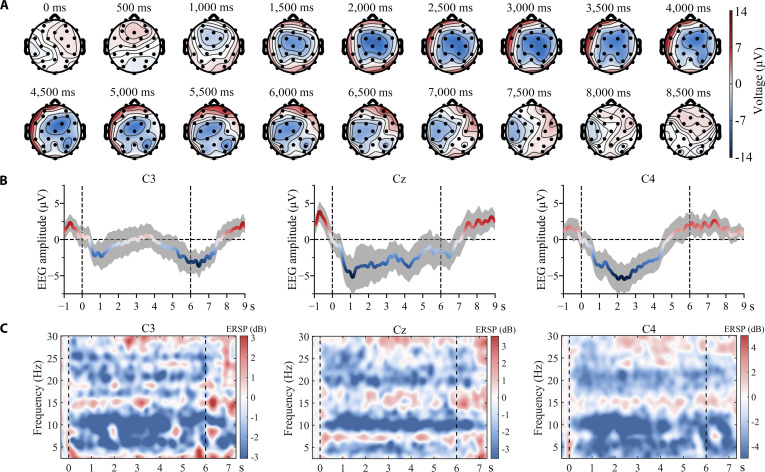
(A) Voltage topography sequence for subject 7. Note that the target starts moving at 1,000 ms and stops when it returns to its original position at 7,000 ms. (B) Averaged MRCPs of electrodes C3, Cz, and C4 for subject 7. Shaded areas represent the 95% CI. (C) Averaged ERSPs of electrodes C3, Cz, and C4 for subject 7. Target movement initiation and termination are marked with vertical dashed lines.

To explain the electrophysiological mechanisms of the paradigm, we plotted a sequence of voltage topographic maps based on the EEG data of 50 trials from one subject, as shown in Fig. 6A. Marked bilateral activation persisted from target movement initiation (1,000 ms) to termination (7,000 ms), demonstrating temporal correspondence with task execution. The activation near the motor cortex (around electrodes C3, Cz, and C4) is particularly pronounced, which aligns with previous findings [43] on brain activation during bilateral motor execution (ME).

In addition to the overall spatial features, we also investigated the responses of individual electrodes. Fig. 6B shows the averaged movement-related cortical potentials (MRCPs) of electrodes C3, Cz, and C4 for subject 7. Before calculating the average MRCP, the data were bandpass filtered between 0.1 and 4 Hz to remove irrelevant frequency components. Marked negative shift began to appear at all 3 electrodes within 1 s prior to movement onset. Fig. 6C shows the averaged event-related spectral perturbation (ERSP) for the same subjects. A notable ERD in the alpha band (8 to 12 Hz) and beta band (12 to 30 Hz) was observed at all 3 electrodes during movement execution. These findings are consistent with our expectations based on previous studies of MRCPs and ERSP [44,45].

## Discussion

This study demonstrated that EEG signals can enable continuous decoding of hand motion angles in polar coordinates during circular tracking movements. The experimental paradigm was validated on 8 subjects using 6 deep learning-based models, including EEGNet, DeepConvNet, ShallowConvNet, EEGNet + LSTM, DeepConvNet + LSTM, and ShallowConvNet + LSTM. The decoding performance was analyzed in terms of MSE, MAE, and CC across 8 subjects. DeepConvNet + LSTM achieved the highest grand-averaged CC, with a value of 0.895 ± 0.053. Its grand-averaged MSE and MAE were (1.012 ± 0.436) rad^2^ and (0.627 ± 0.152) rad. EEGNet + LSTM showed no significant differences from DeepConvNet + LSTM across the 3 metrics. All models exhibited test performance that exceeded their respective chance levels, which indicate the feasibility of continuous angular decoding of circular hand motion in polar coordinates using EEG signals.

Cartesian and polar coordinate systems are commonly used to describe movement. Cartesian coordinates are particularly effective for representing linear trajectories, such as straight-line reaching motions in center-out paradigms [46–48]. Their strength lies in their intuitive alignment with rectilinear dimensions, simplifying the modeling of direction and displacement in Euclidean space. Yet, real-world limb movements encompass far more than straight-line motions, including acts like turning knobs, stirring liquids, and steering vehicles. For these rotational or curvilinear movements, the polar coordinate system may enhance decoding performance by directly representing angular and radial displacement. The angular component inherently captures rotational dynamics, reducing computational complexity in decoding periodic or nonlinear motions. Therefore, this study employed a circular motion paradigm to serve as an appropriate framework for angular decoding while better approximating the aforementioned rotational movement patterns. This approach demonstrates the potential of polar representations to decode complex, real-world movements for practical applications in rehabilitation robotic systems [49–52].

To validate the feasibility of angular decoding across multiple models, this study employed 3 commonly used CNNs, including EEGNet, DeepConvNet, and ShallowConvNet. While these CNNs primarily focus on spatial patterns through their filter-bank architecture and are predominantly used for classification tasks [41,42], this study involved a regression task requiring temporal information processing. Therefore, hybrid architectures combining these 3 CNNs with LSTM layers were adopted to address temporal dependencies. As evidenced by the evaluation results, the hybrid architectures demonstrated varying degrees of superior performance compared to their original CNN counterparts. For example, the average MAE of EEGNet + LSTM is 0.162 rad lower than EEGNet. The LSTM layer effectively leverages the temporal information present in sequential data [53]. By combining CNN and LSTM, more information can be effectively utilized, allowing the hybrid network to achieve better performance than convolutional networks alone. Notably, other temporal models beyond LSTM may also offer distinct advantages in capturing sequential dependencies. For instance, gated recurrent unit (GRU) provides a simplified gating mechanism compared to LSTM, potentially improving training efficiency. The Transformer employs self-attention mechanism, which offers advantages in capturing long-range dependencies. Both models have been applied in EEG decoding, yet primarily for classification tasks [54,55]. Their potential for continuous motion decoding remains to be explored.

Although positive results have been achieved, there were 2 main limitations in this study. Firstly, the fixed radius of the circular trajectory employed in the paradigm diminishes the randomness inherent in actual limb movements. Secondly, this study exclusively employed the ME paradigm, whereas the motor imagery (MI) paradigm is generally more suitable for patients with motor function loss [56,57]. Given that the activation areas differ between MI and ME [58], the decoding performance of MI may be adversely affected. Based upon abovementioned findings and limitations, future research will focus on the following aspects. One primary direction is to develop more complex motor paradigms. This involves shifting from fixed-radius to random-radius trajectories and creating hybrid paradigms that combine circular with linear motions to better mimic naturalistic hand movements. Another key objective involves employing alternative temporal models, such as GRU and Transformers, to further optimize decoding performance. Ultimately, a shift from the ME to the MI paradigm could enhance its clinical relevance for patients with motor function loss.

## Conclusion

In this work, we designed a circular motion paradigm to continuously decode the polar angle of hand in polar coordinates from EEG. Six deep learning models, including commonly used EEGNet, DeepConvNet, and ShallowConvNet, and their variants incorporating LSTM layers, were employed. The decoding results, measured by MSE, MAE, and CC, significantly surpassed chance levels for all models. The best-performing model achieved an MSE of 1.012 rad^2^, an MAE of 0.627 rad, and a CC of 0.895, with values averaged across 8 subjects. These findings demonstrate the feasibility of continuous angular decoding of circular hand motion in polar coordinates using EEG signals. This approach provides a promising alternative to conventional Cartesian-based decoding methods, particularly for applications involving circular or rotational movements.

## Data Availability

All data supporting this study are available upon request.
